# Successfully Managed Toxic Megacolon Due to Clostridium difficile Infection in a Pheochromocytoma Patient Complicated by Cardiogenic and Septic Shock: A Case Report

**DOI:** 10.7759/cureus.72750

**Published:** 2024-10-31

**Authors:** Tinatin Jomidava, Maka Mikadze, Irakli Gogokhia, Magdana Gutashvili, Teona Kakhidze, Nino Sikharulidze, Ana Boqolishvili, Tinatin Makaridze, Tamar Didbaridze

**Affiliations:** 1 Infectious Disease, American Hospital Tbilisi, Tbilisi, GEO; 2 Pharmacology, European University, Tbilisi, GEO; 3 Microbiology, Tbilisi State Medical University, Tbilisi, GEO; 4 Intensive Care Unit, American Hospital Tbilisi, Tbilisi, GEO; 5 Anesthesia and Critical Care, American Hospital Tbilisi, Tbilisi, GEO; 6 Critical Care Medicine, American Hospital Tbilisi, Tbilisi, GEO; 7 Internal Medicine, American Hospital Tbilisi, Tbilisi, GEO; 8 Microbiology, The First University Clinic of Tbilisi State Medical University, Tbilisi, GEO

**Keywords:** adrenal pheochromocytoma, clostridium difficile infection, sepsis, septic shock management, toxic megacolon

## Abstract

In recent years, there has been a significant rise in both the frequency and severity of *Clostridium difficile* colitis. This infection presents a broad clinical spectrum, ranging from asymptomatic colonization to severe fulminant colitis, which often requires urgent surgical intervention. The failure of medical treatments and the development of toxic megacolon typically necessitate surgery, though it is associated with high mortality rates. In this case, we successfully managed a rare instance of fulminant colitis in a pheochromocytoma patient with complicated cardiogenic and septic shock through conservative therapy, utilizing the standard vancomycin-metronidazole combination, supplemented with rifaximin.

## Introduction

*Clostridium difficile* infection (CDI) has become an increasingly prevalent and serious healthcare-associated infection in recent years, with rising incidence and severity, particularly in hospitalized patients or those with recent antibiotic exposure [1,2]. The clinical spectrum of CDI ranges from asymptomatic colonization to mild diarrhea, with more severe cases presenting as fulminant colitis, a life-threatening condition that often requires urgent medical and surgical intervention [3,4].

Fulminant *C. difficile* colitis is characterized by rapid progression, severe inflammation, and the risk of developing toxic megacolon, bowel perforation, and septic shock. In such cases, mortality rates remain alarmingly high, especially when surgery is required, with reported rates ranging from 38% to 80% [5,6]. Standard medical therapy for severe CDI includes oral vancomycin and metronidazole [7]. However, medical management can fail in fulminant cases, necessitating urgent surgical treatment, usually a colectomy [8].

Despite the high mortality and surgical necessity in severe cases [8,9], we report a rare instance of fulminant *C. difficile* colitis that was managed successfully with conservative therapy, avoiding surgery. This case highlights the efficacy of a treatment regimen consisting of vancomycin and metronidazole, supplemented with the addition of rifaximin, a minimally absorbed oral antibiotic that has shown potential in treating recurrent and resistant cases of CDI [10-12].

This report aims to illustrate the potential benefits of incorporating rifaximin into the management of fulminant CDI and explore the factors contributing to successful conservative treatment in a typically life-threatening condition.

## Case presentation

A 55-year-old woman presented to the clinic on 02.05.2024, with complaints of worsening systemic symptoms. The patient reported initially developing flu-like symptoms, including fever, general weakness, cough, runny nose, and loss of taste and smell, approximately one week prior to her hospitalization. These symptoms were consistent with a viral infection. Without seeking medical advice, she self-administered amoxicillin-clavulanic acid for five days. Following this, the fever subsided, though the cough and weakness persisted.

Two to three days before hospitalization, the patient began experiencing abdominal pain, nausea, and vomiting, which were triggered by food intake. She had one episode of diarrhea. On the day of admission, her condition worsened, with new symptoms of chills, a fever of 38°C, and elevated blood pressure at 180/100 mmHg.

Her past medical history includes hypothyroidism, diagnosed 10 years ago, which remains unmanaged. She has not been under the supervision of an endocrinologist and has been inconsistently taking L-thyroxine in varying doses (50 mg or 75 mg). She also has a history of Meniere's disease, diagnosed seven years ago.

At the time of admission, the patient continued to complain of nausea, chills, fever, mental status change, and respiratory distress. Mechanical ventilation was initiated. Further evaluation and management were initiated at the hospital to address her acute and underlying medical conditions.

On admission, the patient's overall condition was severe. Hemodynamic parameters showed a pulse rate of 139 bpm and blood pressure of 135/90 mmHg. The patient remained on mechanical ventilation with selected respiratory parameters, maintaining SpO2 at 98%. Neurological status could not be assessed as the patient was under sedation. Pupils were dilated bilaterally. The patient underwent computed tomography (CT) angiography of the brain, chest, and abdomen. No focal brain pathology was identified, but bilateral areas of lung tissue consolidation were observed, primarily in the lower lobe of the left lung, dorsally, peribronchially, and subpleurally. Additionally, nodular consolidation was noted in the left lingular segments and segments VIII and IX. These findings were consistent with pneumonia. A heterogeneous mass was found in the projection of the lateral leg of the left adrenal gland and the upper pole of the left kidney (pheochromocytoma).

Cardiac troponin I was measured at 7.94 ng/ml (N<0.16). Urgent coronary angiography was not deemed necessary at this stage. The patient was transferred to the intensive care unit (ICU) on 02.05.2024, at 22:30 due to her critical condition. Echocardiography revealed an ejection fraction (EF) of 17%. Neurological status at the time of transfer showed maximally dilated pupils, and the patient was under midazolam infusion. The patient remained on mechanical ventilation with stable hemodynamics most likely due to episodes of pheochromocytoma crisis accompanied by spikes in blood pressure and heart rate, and treatment and monitoring were continued.

Over time, the patient's condition worsened, with unstable hemodynamics requiring maximal doses of pressor support. Troponin levels were monitored, reaching >25 ng/ml. The patient underwent urgent catheterization and coronary angiography, which revealed no vascular pathology. An electroencephalogram (EEG) was performed, showing normal brain activity. The patient experienced episodes of fever and leukocytosis (Table 1), with a white blood cell (WBC) count of 41, showing a rising trend with neutrophilia and a left shift in the differential. At this point, the working clinical diagnosis was as follows: pneumonia, pheochromocytoma, respiratory failure, sepsis, adrenal crisis, and combined cardiogenic and septic shock with an EF of 17%. Antibiotic therapy was adjusted to maximal doses of meropenem and vancomycin.

**Table 1:** Laboratory parameter changes before, during, and after the conservative treatment WBC: white blood cell; CRP: C-reactive protein; PCT: procalcitonin test; CREA: creatinine; ALT: alanine aminotransferase; AST: aspartate aminotransferase; ALB: albumin; LDH: lactate dehydrogenase; EF: ejection fraction; GDH: glutamate dehydrogenase

	02.05.2024	17.05.2024	24.05.2024	29.05.2024	Normal values
WBC	41	21	14.36	11.15	4.5-11 10^9^/L
CRP	50	69	57.26	7.10	5 mg/L
PCT	0.8	1	1.5	0.3	0.5 ng/mL
CREA	75	125	63	57	45-84 µmol/L
Urinalysis	N	N	N	N	N
Troponin I	7.94	25	2.3	0.8	0.16 ng/mL
ALT	116	130	42.8	26	33 U/I
AST	114	140	38.8	29	32 U/I
ALB	32.9	28	37.1	37.6	39.7-49.4 g/L
LDH	340	380	487	213	135-214 U/I
EF	17%	54%	55%	54%	54-74%
*Clostridium difficile* GDH	N/A	+	+	+	-
*Clostridium difficile* toxins	N/A	A(+) B(+)	A(+) B(+)	A(-) B(-)	-
Metanephrine concentration in urine	11841 ng/L	-	-	-	-
Metanephrine in 24-hour urine	29603 ng/24	-	-	-	43.4-260 ng/24
Normetanephrine in 24-hour urine	10290 ng/24	-	-	-	128-484 ng/24
Normetanephrine concentration in urine	4116 ng/L	-	-	-	-

The patient's condition showed relative improvement, and pressor support was discontinued. However, intermittent episodes of pheochromocytoma crisis persisted, and treatment with alpha and beta blockers was continued. Sedation was stopped on 06.05.24, and the patient became responsive and followed simple commands, although there was delayed motor activity in all four limbs, more pronounced in the lower limbs. By 12.05.24, respiratory parameters were satisfactory on room air, and a chest X-ray showed no radiological signs of pneumonia. Extubation was performed in accordance with the protocol.

The patient's condition remained concerning. Neurological status showed clear consciousness, and motor function in the limbs improved, but episodes of pheochromocytoma crisis continued, marked by severe tachycardia, hypertension, nausea, and vomiting. The doses of alpha and beta blockers were adjusted accordingly. Echocardiography was performed in dynamics, showing an EF of 54%. Blood tests revealed leukocytosis with a WBC count of 21. Pneumonia was ruled out through a chest X-ray, and no urinary tract infection was found in the urinalysis. The central venous and bladder catheters were removed.

On 16.05.24, the patient experienced abdominal bloating, nausea, and vomiting, which were not related to blood pressure spikes, and no bowel movement was observed. The patient didn't have any bowel movements for 72 hours. After minimal bowel movement, stool samples were sent for *C. difficile* testing. The results came back positive for *C. difficile* toxins A and B and glutamate dehydrogenase. The highest recommended dose of oral vancomycin treatment was initiated. The patient's condition worsened, with progressive respiratory failure and uncorrected respiratory alkalosis due to high abdominal pressure.

On 17.05.24, an abdominal CT was performed, ruling out acute surgical pathology: Repeated CT scans show elevated positioning of the right diaphragm dome. A small amount of fluid is observed in the pericardial cavity.

In the projection of the left adrenal gland and the upper edge of the left kidney, in individual slices, a mass with unclear contours and heterogeneous structure is visualized, with a Hounsfield unit (HU) of 110, with a maximum size of 5.5×5.7 cm.

**Figure 1:**
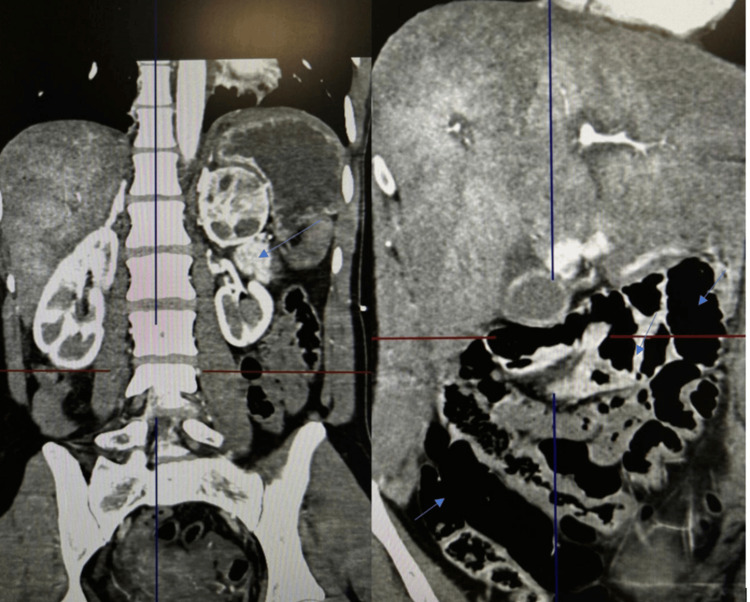
A heterogeneous mass was in the projection of the lateral leg of the left adrenal gland and the upper pole of the left kidney. The stomach, small intestine, and large intestine up to the level of the sigmoid colon were unevenly and markedly dilated, with thickened walls, and were filled with gas and contents. Horizontal fluid and air levels are seen in some areas. Dense contents are observed in the lumen of the cecum and ascending colon. Hyperdense areas are visualized in the projection of the duodenal bulb. Paralytic ileus secondary to CDI was suspected CDI: *Clostridium difficile* infection

The sigmoid colon was elongated and formed a loop (dolichosigma). The stomach, small intestine, and large intestine up to the level of the sigmoid colon were unevenly and markedly dilated, with thickened walls, and were filled with gas and contents (Figure 1). Horizontal fluid and air levels are seen in some areas. Dense contents are observed in the lumen of the cecum and ascending colon. In the projection of the duodenal bulb, hyperdense areas are visualized, measuring 1.2 cm. Paralytic ileus secondary to CDI was suspected.

Small amounts of fluid were seen subdiaphragmatically, adjacent to the lower edges of the liver and spleen, in some inter-intestinal spaces, and in the pelvic cavity.

Due to worsening respiratory failure, the patient was placed back on mechanical ventilation on 17.05.24 with pressor support initiated for unstable hemodynamics. Blood tests revealed anemia with hemoglobin at 62 g/L, prompting blood transfusion. Electromyography of all four limbs and a nerve conduction study of both diaphragmatic nerves were conducted under deep sedation, revealing a sensorimotor axonal polyneuropathy with muscle denervation, categorized as critical illness polyneuropathy. The main antimicrobial therapy was modified. Metronidazole 500 mg every eight hours was added to existing maximal doses of oral vancomycin (500 mg every eight hours) plus rectal vancomycin.

In the following days, the patient's condition improved, and respiratory parameters were simplified. The patient was extubated again after meeting the criteria for weaning. No further episodes of pheochromocytoma crisis were noted, and the fever subsided. Inflammatory markers showed improvement. However, the patient exhibited signs of paralytic ileus with mucus-like stool secretions. A surgical consultation was conducted to assess the management of paralytic ileus and megacolon. Despite conservative measures, bowel function did not fully recover.

From a surgical standpoint, at this stage, operative treatment was not considered necessary. An abdominal CT with oral contrast was planned to assess bowel passage.

**Figure 2:**
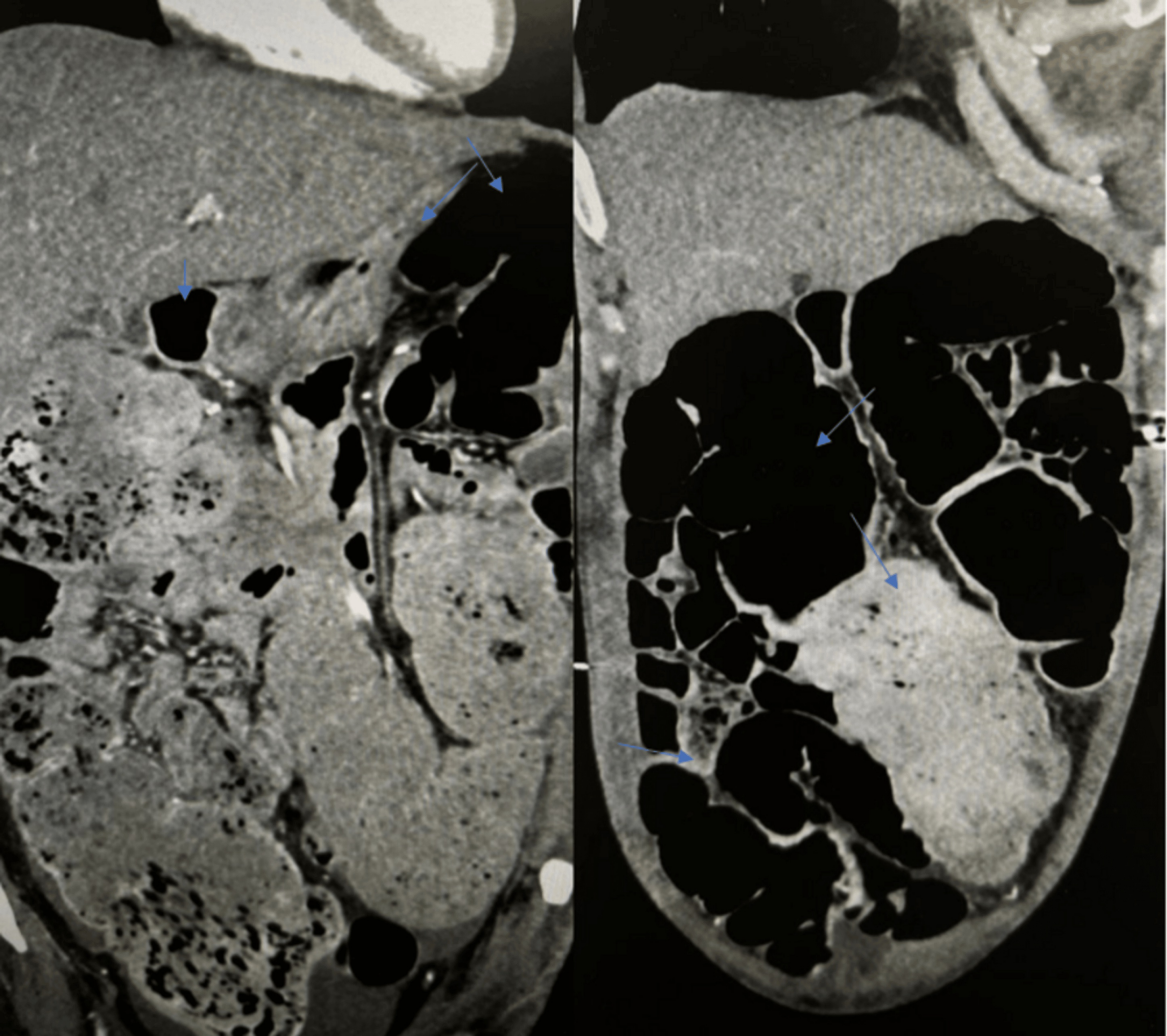
The cecum, ascending colon, and transverse colon along their entire length, as well as the sigmoid colon at the loop level, are unevenly and markedly dilated, filled with gas and contents. The degree of large intestine dilation is increased compared to the previous study. The cecum is somewhat ptotic, displaced medially, compressing the uterus and urinary bladder

Repeated CT scans (24.05.24) show elevated positioning of the right diaphragm dome. The cecum, ascending colon, and transverse colon along their entire length, as well as the sigmoid colon at the loop level, are unevenly and markedly dilated, filled with gas and contents. Horizontal fluid and air levels were seen in some areas. The degree of large intestine dilation is increased compared to the previous study (Figure 2). The cecum is somewhat ptotic, displaced medially, compressing the uterus and urinary bladder.

Small amounts of free fluid are seen subdiaphragmatically on both sides, adjacent to the lower edges of the liver and spleen, in some inter-intestinal spaces, and in the pelvic cavity.

A medical board meeting consisting of the surgeon, the medical director, the head of the ICU department, the radiologist, the endocrinologist, and the infectious disease physician was conducted. It was decided that because of the high risks, surgical intervention would only be considered in urgent cases (such as bowel perforation, necrosis, or acute peritonitis). The main antimicrobial therapy was modified, and rifaximin (1200 mg/d) was added to the regimen. With ongoing conservative measures, bowel motility was restored, and regular bowel movements occurred.

On 29.05.24, stool samples tested negative for *C. difficile* toxins, and the patient's general condition stabilized. The patient became more active. Hemodynamics remained stable, with sinus rhythm and compensated respiratory status on room air. The central venous and bladder catheters were removed. Lab results showed the following: WBC count 11.1, neutrophils 75%, hemoglobin 8.5 g/L, CRP 7.1 mg/L, and platelets 265. The patient was transferred to the internal medicine department for further evaluation and treatment.

The patient continued to receive cardiopulmonary monitoring, crystalloids, and symptomatic therapy with close endocrinology follow-up. Following treatment, the patient's condition improved both clinically and in laboratory findings. At this stage, inpatient treatment was no longer necessary, and the patient was discharged home with outpatient follow-up recommendations including abdominal CT and further planned surgery for pheochromocytoma treatment.

## Discussion

This case illustrates a rare and complex presentation of fulminant CDI in a patient with a history of pheochromocytoma, complicated with cardiogenic and septic shock, which was successfully managed through conservative therapy.

The case highlights several important aspects in the management of severe CDI, the role of appropriate antibiotic use, and the challenges of balancing comorbidities in critically ill patients. The patient's underlying condition of pheochromocytoma contributed to the complexity of management. Pheochromocytoma led to episodes of hypertensive crises, tachycardia, and other adrenergic symptoms, further complicating her critical state. This condition required specialized management with alpha and beta blockers, which needed to be closely monitored due to the risk of exacerbating hemodynamic instability. The careful titration of these agents and ongoing endocrine supervision were crucial in stabilizing the patient's condition during her recovery from the CDI. Fulminant CDI is a life-threatening condition that often requires immediate surgical intervention, especially in cases of toxic megacolon, bowel perforation, or necrotizing colitis. Surgical mortality rates for patients with fulminant CDI are high, ranging from 38% to 80% [1], making timely decision-making critical.

In this patient, surgery was initially considered due to the severe symptoms and risk of bowel necrosis and perforation. However, a multidisciplinary team decided to opt for aggressive conservative management given the patient's high surgical risk and her already fragile state due to cardiogenic and septic shock. Conservative treatment was initiated with a standard combination of oral and rectal vancomycin and intravenous metronidazole, both cornerstones in the treatment of severe CDI [7,13].

Rifaximin, a minimally absorbed oral antibiotic often used for recurrent or refractory cases of *C. difficile*, was added to the regimen. Rifaximin has been shown to help reduce the recurrence of CDI [11,14] and improve outcomes in resistant cases due to its ability to target *C. difficile* in the gut without significant systemic absorption [15]. In this case, the conservative approach proved effective, with gradual improvement in the patient's bowel function and resolution of *C. difficile* toxin positivity in stool samples by day 29 of hospitalization.

The decision to avoid surgery in favor of a medical approach underscores the potential efficacy of intensive antibiotic therapy in select cases of fulminant CDI, particularly when complicated by severe comorbidities. Throughout the patient's ICU stay, her condition fluctuated with periods of hemodynamic instability, respiratory failure, and critical illness polyneuropathy, all of which were managed with a combination of mechanical ventilation, pressor support, and transfusions as needed.

Regular imaging and clinical monitoring were essential in determining that no acute surgical pathology, such as bowel perforation, had developed, allowing the team to pursue nonoperative treatment. This approach not only avoided the high risks associated with surgery but also allowed time for the patient's condition to stabilize with medical therapy.

## Conclusions

This case demonstrates the potential for the successful conservative management of fulminant CDI, even in high-risk patients with significant comorbidities. The key to this successful outcome was the comprehensive, multidisciplinary approach to care, the judicious use of antibiotics, and the careful monitoring of both infection and the patient's underlying conditions. This case also highlights the importance of avoiding unnecessary antibiotic use, highlighting the need for antibiotic stewardship in preventing severe healthcare-associated infections. Further attempts should be made to aid in the early diagnosis of the infection and prompt management of the patient. In addition, more high-quality research is needed to develop and standardize new alternative treatment regimens for cases unresponsive to standard treatment.
